# Screening of Ten Tomato Varieties Processing Waste for Bioactive Components and Their Related Antioxidant and Antimicrobial Activities

**DOI:** 10.3390/antiox8080292

**Published:** 2019-08-08

**Authors:** Katalin Szabo, Zorița Diaconeasa, Adriana-Florinela Cătoi, Dan Cristian Vodnar

**Affiliations:** 1 Institute of Life Sciences, University of Agricultural Sciences and Veterinary Medicine, 400372 Cluj-Napoca, Romania; 2 Department of Food Science and Technology, University of Agricultural Sciences and Veterinary Medicine, 400372 Cluj-Napoca, Romania; 3 Pathophysiology Department, “Iuliu Haţieganu” University of Medicine and Pharmacy, 400000 Cluj-Napoca, Romania

**Keywords:** tomato peels, revalorization, carotenoids, phenolic compounds, antioxidant, bioactive

## Abstract

Global tomato production is currently around 180 million tons, of which more than a quarter undergoes processing. The removed peels, seeds, and vascular tissues usually end up in landfills, creating environmental pollution. In order to highlight the alternative use of these vegetal wastes, our study investigated 10 tomato varieties in terms of carotenoids content, phenolic composition, and their related antioxidant and antimicrobial activities. Tomato peels extracts were screened by high performance liquid chromatography with diode-array detection (HPLC/DAD) for qualitative and quantitative analyses. The extracts were tested against six bacterial strains to determine their antimicrobial effect; the 1,1-diphenyl-2-picrylhydrazyl (DPPH) assay was applied to estimate their antioxidant capacity. Total carotenoids content was significantly higher in *Ţărănești roz*, a local variety (5.31 ± 0.12 mg/100 g DW), while *Mirsini*, a commercial hybrid, presented significantly higher total phenolic content (155 ± 2 mg/100 g DW) compared to the mean value of all analyzed samples. The methanolic extracts of tomato peels presented acceptable antimicrobial activity against *Staphilococcus aureus* and *Bacillus subtilis*, and the mean antioxidant activity was 201 ± 44 µmol Trolox/100 g DW tomato peels. Considering that tomato peels have lycopene, β-carotene, lutein, and different phenolic compounds in their composition, tomato industrial by-products could represent a source of natural bioactive molecules with applicability in nutraceuticals and food industry.

## 1. Introduction

Tomatoes (*Solanum lycopersicum*) are one of the most popular vegetables worldwide with an annual production of approximately 180 million tons above (FAOSTAT, 2017). Due to their content in bioactive components, tomatoes and their consumption are linked to important health benefits, like improving heart health and preventing some oxidative stress-related diseases [1]. 

The main production regions are located in temperate zones; therefore, the crop has a seasonal trend, most of the tomatoes being processed between the months of July and December. Tomato processing results in various food products like sauces, canned tomatoes, ketchup or juice. On the other hand, it generates important quantities of by-products. About a quarter of the total tomato production undergoes processing, which means that tomato peels, seeds, and small amounts of pulp are removed during treatments. These by-products can sum up to 5–30% of the main product, and are used as livestock feed or discarded in landfills, creating serious environmental problems [2].

According to Kalogeropoulos and co-authors [3], the phytochemicals found in industrial tomatoes and their by-products include carotenoids, polyphenols, tocopherols, some terpenes, and sterols. These bioactive molecules seem to resist industrial treatment, nominating tomato processing wastes as a source of natural bioactive molecules.

Carotenoids are organic pigments found in plat species, some algae and fungi, conferring the yellow, orange, and red colors to the producing organism. They are known to improve human health by antioxidant activities, enhancement of the immune system together with the reduction of the risk of degenerative diseases such as cancer, cardiovascular diseases, cataract and macular degeneration [4]. Recent studies highlight that women with higher plasma concentrations of β-carotene and α-carotene are at lower breast cancer risk of ER2 breast cancer [5]. Furthermore, lycopene supplementation was found effective against aging-related inflammatory and oxidative stress-induced neurodegeneration [6] and (13Z)-lycopene could be used as an indicator of oxidative damage to lycopene in smokers [7]. Besides these functions, carotenoids have provitamin A activity and can be incorporated as bioactive ingredients in food formulations to improve the final products’ shelf life and sensory properties [8].

Tomatoes contain high amounts of carotenoids, mostly lycopene, β-carotene and lutein. Numerous extraction methods are available to recover these phytochemicals from the tomato processing waste; however, the yields may vary, depending mostly on the tomato variety and on the industrial processing methods along with the solvents used in the extraction protocols and the parameters applied [4]. Ultrasound-assisted extraction (UAE) is a promising method, because of the advantages including: (i) an overall enhancement of extraction yield of heat-sensitive bioactive compounds by enabling lower processing temperatures; (ii) the opportunity to use alternative “generally recognized as safe” (GRAS) solvents by improvement of their extraction performance; and (iii), the reduction of processing time *vs.* conventional extraction. The extraction yield of carotenoids raised up to 143%, due to the ultrasound treatment used on tomato by-products, and caused no degradation of the carotenoids, compared to the conventional extraction methods [9]. Another green technique applied to extract carotenoids, or other bioactive components, from different vegetal matrices is supercritical fluid extraction which usually operates with GRAS solvents or no organic solvents, in some cases [10]. Furthermore, according to Yara-Varon et al., cyclopentyl methyl ether and 2-methyltetrahydrofuran could be used as alternative green solvents for extracting carotenoids [11].

Phenolic compounds are a large group of plant secondary metabolites which gained increasing preoccupation recently because of the growing body of evidences indicating the positive effects of plant-derived phenolics on the prevention or the initiation of a large variety of diseases [12,13]. In a recent study, different fruit and vegetable by-product’s flour was incorporated to spreadable cheese and the fortified final product presented significantly higher phenolic and flavonoid contents related to the control sample [14]. Phenolic compounds found in tomato wastes are phenolic acids (caffeic, chlorogenic, p-coumaric, ferulic and rosmarinic acid) and flavonols (quercetin and rutin), as reported by Ćetković et al. [15]; their results suggest that tomato waste should be regarded as potential nutraceuticals resource, based on the significant antioxidant and antiproliferative activities of the extracts. Phenolic compounds can also be recovered by UAE as the ultrasound waves help to disrupt plant cell walls and can improve the solvent penetration, thus, enhancing mass transfer across the cell membrane [16].

In our previous study (in press) we investigated the bioactive and antioxidant properties of extracts from several tomato varieties’ processing wastes (seeds and pomace) in order to examine how bioactivity relates to their composition. The aim of the present study is to evaluate the phenolic compounds and carotenoids content of a different fraction, namely tomato peels, of ten tomato varieties processing waste through UAE and to determine the antioxidant and antimicrobial capacities of the extracts.

## 2. Materials and Methods

### 2.1. Chemicals and Standards

Acetic acid, acetonitrile, methanol, petroleum ether, ethyl acetate, cyanidin chloride, DPPH, Trolox and other reagents implied in the experiments were of analytical grade, purchased from Sigma-Aldrich (Steinheim, Germany); carotenoid standards (β-carotene), as well as chemicals used for antimicrobial assays, Mueller-Hinton agar and Mueller-Hinton broth, peptone special, triptic soy broth, starch, resazurin, were purchased from BioMerieux (Marcy l’Etoile, France).

### 2.2. Sample Preparation

Ten tomato varieties, with the same provenience (Horticulture Research and Development Centre, Cluj-Napoca, Romania) and cultivated similarly, were used in the present study: six international cultivars (*Abellus, Aphen, Cristal, Misrini, Lady Rosa, Tiny Tim*) and four local varieties (*Ţărănești roz, Ţărănești portocalii, Rotunde mari,* and *Roșii lunguieţe*). Tomato fruits were harvested in August 2018, when more than 90% of the fruits surface was red (color classification according to USDA, 2005). The samples (5 kg tomatoes from each variety) were washed, cut in pieces and tomato juice was obtained by a manual tomato juicer. The resulted by-product was separated in two fractions (peels and seeds) by decantation. Tomato peels were dehydrated for 48 h in the dark, to avoid carotenoids loss, using a food dehydrator (Heinner HFD-404TD, Bucuresti, Romania) at 38 °C. Dried samples were grounded into powder using an analytical mill (A 10 basic IKA, Sartorom, Romania) and after passing through sieve of mesh number 10, powders having particle sizes <2 mm were separated for next usage and stored in brown paper bags in the dark at room temperature until further analysis. The dry matter of the tomato peels was determined gravimetrically using oven-drying at 105 °C until a constant weight.

### 2.3. UAE of Carotenoids

Carotenoids were extracted from the tomato peels applying the protocol described by Bunea et al. [17]. Briefly, a mixture of methanol/ethyl: acetate/petroleum: ether (1:1:1, *v*/*v*/*v*) was used to extract total carotenoids from each powder sample (1 g). Falcon tubes containing the sample together with 10 mL solvent was placed in an ultrasonic unit (Elma Schmidbauer GmbH, Singen, Germany) for 10 min, centrifuged at 11,000 RPM and filtrated. The remained residue was re-extracted two more times by applying the same protocol. The extracts were collected in a separation funnel and were successively washed with sodium chloride solution (15%) and diethyl ether. The organic phase (upper layer), enclosing the targeted carotenoids, was dried over anhydrous sodium sulphate and the solvent was removed by a rotary evaporator (Rotavapor R-124, Buchi, Flawil, Switzerland) at 35 °C.

### 2.4. Quantitative and Qualitative Analysis of Carotenoids (Lycopene, β-carotene and Lutein) by HPLC/DAD

The extracts were dissolved in 1 mL ethyl acetate, filtered through a Millipore filter with 0.45 µm pore size and injected into the HPLC/DAD system. Individual carotenoids, lycopene, β-carotene and lutein, were detected by a diode array detector couplet to an Agilent 1200 HPLC system (Agilent Tehnologies, Santa Clara, CA, USA) with a high purity reversed phase Nucleodur C18 ec column (Macherey-Nagel, Düren, Germany). Mobile phase A was a mixture of acetonitrile:water (9:1, *v*/*v*) with 0.25% trimethylamine and mobile phase B was ethyl acetate with 0.25% trimethylamine, eluted with a flow rate of 1 mL/min. The chromatograms were observed at 450 nm wavelength and the HPLC peaks were identified using carotenoid standards (lycopene, β-carotene and lutein). Quantification of carotenoids was made by using the calibration curve of the β-carotene standard. Total carotenoids content was expressed as the sum of individual carotenoids (mg β-carotene/100 g DW tomato peels).

### 2.5. UAE of Phenolic Compounds

The phenolic compounds were extracted using a method described previously by Choi et al. [18]. A sample of 0.5 g of tomato peels powder was placed into a 25 mL Falcon tube and brought up to volume with 80% methanol in water. The container with the sample was ultra-sonicated for 60 min at 30 °C, and centrifuged at 18,000× *g* for 10 min, at 1 °C. The supernatant was filtered through a Millipore filter with 0.45 µm pore size and the obtained filtrate was used for qualitative and quantitative analysis of phenolic compounds and for assaying antioxidant and antimicrobial capacities.

### 2.6. Qualitative and Quantitative Analysis of Phenolic Compounds by HPLC-DAD-ESI-MS

The phenolic compounds were determined by a high performance liquid chromatograph with diode array detection and electrospray ionization mass spectrometry (HPLC-DAD-ESI-MS) using an Agilent 1200 HPLC system (Agilent Tehnologies, Santa Clara, CA, USA) equipped with an Eclipse column, XDB C18 (4.6 × 150 mm, 5 mm). The mobile phases, solvent A, consisted of 0.1% acetic acid:acetonitrile (99:1) in distilled water (*v*/*v*), and solvent B, consisted of 0.1% acetic acid in acetonitrile (*v*/*v*), were eluted at a flow rate of 0.5 mL/min, following a previously used elution program [19]. 

The phenolic compounds were identified on the basis of their retention times, comparing to reference standards. The MS fragmentation was performed at a capillary voltage of 3000 V with a scanning range situated between 100 and 1000 *m*/*z*, at 350 °C and a nitrogen flow rate of 8 L/min. The data was analyzed by Agilent ChemStation Software (Rev B.04.02 SP1, Palo Alto, CA, USA). Total phenolic content was calculated as the sum of individual concentrations of the phenolic components. 

### 2.7. Antimicrobial Activities

#### 2.7.1. Bacteria and Culture Conditions

For this assay, we used three Gram-positive bacterial strains: *Staphylococcus aureus* (ATCC 49444), *Bacillus subtilis* (ATCC 11778) and *Listeria monocytogenes* (ATCC 19114); and three Gram-negative strains: *Escherichia coli* (ATCC 25922), *Pseudomonas aeruginosa* (ATCC 27853) and *Salmonella typhimurium* (ATCC 14028). All strains involved in the testing of the antimicrobial capacities of the tomato peels extracts were obtained from the Food Biotechnology Laboratory of our university. Bacteria were stored at 4 °C and sub-cultured monthly on Mueller-Hinton (MH) agar.

#### 2.7.2. Micro-Dilution Method

To evaluate the antimicrobial capacity of the tomato peels extract, the modified micro-dilution technique was applied, previously described by Vodnar et al. [20]. Concisely, fresh overnight cell suspensions were adjusted to a concentration of approximately 2 × 10^5^ colony forming units (CFU)/mL, with sterile saline solution, in a final volume of 100 µL per well.

We performed minimum inhibitory concentrations (MICs) determinations using serial dilutions in 96-well plates. Two-fold diluted samples were placed in wells with 100 µL of MH broth and 10 µL of inoculum. We used methanol (80%) in water as control. Afterwards, the microplates were incubated at 37 °C for 24–48 h. Next, we added 20 µL (0.2 mg/mL) of resazurin solution to each well, followed by a 2 h incubation at 37 °C. Reduction of resazurin (color change from blue to pink) indicates bacterial growth. MICs were determined as the lowest concentration of samples (mg/mL) which inhibited bacterial growth *vs*. control.

### 2.8. Antioxidant Activity

DPPH (1,1-diphenyl-2-picrylhydrazyl) free radical scavenging capacity of the tomato peels extracts was evaluated spectrophotometrically by a slightly modified method of Brand-Williams, Cuvelier, and Berset [21] as described by Dulf et al. [19,22]. Briefly, methanolic extract/standard solution (40 μL) was mixed with 200 μL of DPPH solution (0.02 mg/mL) and incubate for 15 min at room temperature and then the absorbance was measured at 517 nm using a multi-mode plate reader (BioTek, Winuschi, VT, USA). The results were expressed as micromol Trolox equivalents (μmol TE) /100 g sample (DW of tomato peels).

### 2.9. Statistical and Multivariate Data Analysis

Carotenoids and phenolic composition of the tomato peels extracts and the associated antioxidant activities were investigated in triplicate, and are presented as mean +/− standard deviation (SD). The variation in bioactive constituents of the studied tomato varieties was analyzed by ANOVA and the differences in their antioxidant capacities and total carotenoids content were examined by the Tuckey’s test (*p* < 0.05). Furthermore, Pearson’s correlation coefficient was used to inspect the relationship between the biological activities (antioxidant and antimicrobial capacities) and carotenoids and phenolic content of the samples. 

## 3. Results and Discussion

### 3.1. Carotenoids Content

#### 3.1.1. Total Carotenoids Content 

Total carotenoids content of the analyzed samples is presented in the last column of Table 1; the values ranged between 0.60 ± 0.05 and 5.31 ± 0.12 mg β-carotene /100 g DW tomato peels. Different letters indicate significant differences by Tukey’s test, at 5% probability. 

The mean carotenoids content of the samples was 2.50 ± 1.29 mg β-carotene/100 g DW. The highest carotenoids content was found in the local variety *Ţărăneşti roz* (5.31 mg β-carotene /100 g DW tomato peels), with significantly higher amounts of total carotenoids compared to all the other samples. 

These findings are in line with previous results reported by Strati, Gogou and Oreopoulou [23], who experimented extraction of total carotenoids from tomato waste with different solvents and found values between 0.36 and 16.52 mg/100 g DW. Strati and Oreopoulou [4] summarized in a review that many factors have impact on the carotenoid’s extraction yields such as tomato processing technology, by-product fragment and/or the extraction methods and parameters applied. Consequently, the variation of carotenoids content in the analyzed samples could be attributed to the tomato variety, which seems to have a compelling influence in the total carotenoids content of the peels. 

Carotenoids intake is important for human health and, in the context of circular economy, revalorization of carotenoids from unexplored natural sources, like tomato processing by-products, could be a solution to ensure proper diet to the population in developing countries. 

#### 3.1.2. Lycopene, β-Carotene and Lutein Content

The individual results for lycopene, β-carotene and lutein are presented in Table 1. Lycopene was found in highest amounts in *Ţărăneşti roz* local variety (3.70 ± 0.02 mg/100 g DW tomato peels), followed by *Rotunde mari* (2.80 ± 0.04 mg/100 g DW). β-carotene content was highest in *Cristal* commercial hybrid (0.59 ± 0.02 mg/100 g DW) and *Ţărănești roz* variety (0.53 ± 0.01 mg/100 g DW). Lutein content was highest in *Ţărănești roz* (1.09 ± 0.09 mg/100 g DW). 

Previous studies on tomato waste extracts reported similar lycopene content ranging between 0.639 and 1.98 mg/100 g [24]. β-carotene content of the analyzed samples was slightly smaller compared to the results found by Shi et al. [25], this fact might be explained by the solvents used for the extraction or the different extraction method applied. Lutein levels of tomato by-products ranged from 9.90 to 10.05 µg/g, as reported by Montesano et al. [26], our results being in line with these findings. At present, the most common source of natural lutein is represented by marigold flowers, however the extraction procedure involves high costs and prolonged development [26], therefore tomato processing by-products may be pointed out as an alternative commercial source of lutein for food functionalization and/or nutraceutical.

### 3.2. Phenolic Compounds Found in Tomato Peels

#### 3.2.1. Total Phenolic Content

Total phenolic contents of the analyzed samples were calculated as the sum of individual phenolics from each sample and are presented in the last column of Table 2. It has to be mentioned that the one-step extraction protocol of the phenolic compounds may cause underestimation of the total content of phenolic compounds in the tomato peel. The values ranged between 37 ± 2 and 155 ± 2 mg/100 g DW, with a mean value of 76 ± 4 mg/100 g DW. Different letters indicate significant differences by Tukey’s test at 5% probability. The multivariate analysis showed significantly higher total phenolic content in *Mirsini* commercial hybrid compared to the other analyzed samples.

Earlier results on the valorization of phenolic composition of 6 tomato varieties waste, reported a total phenolic content ranging between 179 and 521 mg/100 g DW [15]. However, these results were obtained on tomato wastes containing peels together with seeds. The total phenolic content from our study is comparable to the values summarized by Choi et al. [18], who recorded total phenolic content ranging between 64.6 and 440.0 mg/100 g DW in cherry tomatoes. The difference between previous results and our findings could be explained by the effect of decantation procedure used to separate the seeds from the peels, which seems to influence the hydrophilic phenolics content. Plant-derived phenolic compounds and their associated biological activities became a popular subject by virtue of the increasing evidences showing protective action against numerous non-communicable human diseases [13,27]. Therefore, tomato processing by-products can provide some functional ingredient in new food formulations.

#### 3.2.2. Individual Contents of Phenolic Compounds

Quercetin-triglucoside (QTG), quercetin-3-rutinoside (or rutin, Q3R), 3,4-di-O-caffeoylquinic (isochlorogenic) acid (di-CQA), 3,4,5-tri-caffeoylquinic acid (tri-CQA), naringenin chalcone (NGC) and naringenin (NG) were identified by HPLC-DAD-ESI-MS from the methanolic extracts of tomato peels. Individual concentrations of the phenolic compounds are presented in Table 2. 

NGC was the main component in majority of the samples, varying between 6.6 ± 0.7 and 87.6 ± 1.2 mg/100 g DW, with a mean value of 34.9 ± 28.5 mg/100 g DW tomato peels. The other predominant compound was Q3R, found in highest values in *Mirsini* commercial hybrid (51 ± 0.1 mg/100 g DW).

Previous results on individual phenolic compounds – identified in tomato peels fiber – recorded Q3R and NG as the main components of the phenolic profile, with values situated between 10.71 and 41.01 mg/100 g for Q3R, and 7.94 and 28.76 mg/100 g for NG [28]. However, the variation in phytochemicals content of tomatoes depends on geographical site of production, variety, ripeness and processing aspects [29,30]. Additionally, large differences in composition can be attributed to genetic factors [18]; these facts could explain the variation of phenolic compounds among the analyzed samples.

### 3.3. Antimicrobial Activity

Tomato peels extracts were subjected to antimicrobial activity against six bacterial strains: three Gram-positive and three Gram-negative microorganisms. The results are presented in Table 3. All extracts presented acceptable antimicrobial activity. The most effective extract was found to be *Ţărăneşti roz* local variety against *S. aureus* and *B. subtilis* (MIC: 2.5 mg tomato peels/mL). A possible explication for this could be the chemical composition of *Ţărăneşti roz*, which contains significantly higher amounts of carotenoids than the other analyzed samples.

Earlier studies regarding antimicrobial capacity of tomato industrial by-products indicate that extracts obtained with different solvents are active only against Gram-positive bacteria [31]. Our findings relate to this trend, with MIC values of 10 to 2.5 mg tomato peels/mL; however, *Abellus, Aphen* and *Cristal* varieties presented antimicrobial effect against *E. coli* with a MIC of 5 mg tomato peels/mL.

Other studies, concerning antimicrobial activity of tomato, reported that *Pitenza* variety extracts inhibited the growth of pathogens such as *E. coli* O157:H7, *S. typhimurium*, *S. aureus*, and *L. ivanovii*, with MIC values of 12.5 to 3.125 mg/mL [32]. In addition, tomato pomace extract slowed down discoloration of lamb meat during 7 day storage, which could positively influence consumer acceptance [33]. Therefore, tomato processing wastes could represent a low-priced antimicrobial agent implied in food packaging and storage.

### 3.4. Antioxidant Activity

**Figure 1:**
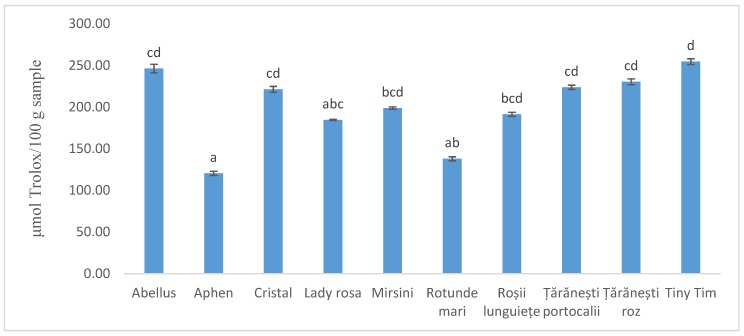
Antioxidant activity of tomato peels extracts by DPPH assay. Different letters indicate significant differences by Tukey’s test, at 5% probability.

The results of DPPH assay are presented in Figure 1. All the tested samples had good antioxidant capacity, except for *Aphen* variety, which presented significantly lower antioxidant capacity compared to the other analyzed samples.

The values ranged between 120 ± 2 and 255 ± 3 μmol TE /100 g sample, with a mean value of 201 ± 44 μmol TE /100 g. The *Tiny Tim* variety revealed the highest antioxidant activity. This fact might be attributed to the synergistic interactions between bioactive components [34], and/or to the cherry type variety, which contains a complex mixture of nutrients and phytochemicals compared to other tomato genotypes, despite their smaller fruit size [35].

Earlier results regarding antioxidant capacity of tomato peels fiber showed different values for the hydrophilic extract (3.90 μmol TE/g) and for the lipophilic extract (0.044 μmol TE/g) and these values were lower than expected [28]. The authors explained the results as a consequence of the high content of insoluble dietary fiber of tomato peels, which entrapped the main phenolic compounds. 

The correlation between bioactive compounds and antioxidant properties is the subject of several research works conducted on fruits and vegetables, however, the antioxidant activity might not always correlate with the amount of phenols [36]. In the present study, Pearson’s correlation coefficient (*r*) showed a weak linear positive relationship (*r* = 0.312) between the total phenolic content and the antioxidant capacity of the samples. 

According to Ćetković et al. [15], the antioxidant capacty of tomato waste extracts cannot be directly associated with a specific phenolic compound, instead it could be connected to the mutual interactions of all hydrophillic antioxidants and other constituents of the tomato waste extracts. Our results support this approach through Pearson’s correlation coefficient, which shows a moderate uphill relationship (*r* = 0.518) between the lutein content of the samples and the antioxidant activity.

## 4. Conclusions

The present study evaluated the bioactive composition of tomato peels obtained after processing separately several tomato varieties. Although each variety was cultivated and processed under identical conditions, the results indicate significant differences. *Ţărăneşti roz* local variety had the highest carotenoids content and was the most efficient against Gram-positive bacteria. *Mirsini* commercial hybrid presented three folds higher rutin content and significantly higher total phenolic content compared to the other analyzed samples. The results highlight that variety has a compelling influence regarding bioactive composition of tomato peels. 

The methanolic extracts of tomato peels presented acceptable antimicrobial activity against *S. aureus* and *B. subtilis* strains, and some varieties were effective against *E. coli* as well.

The antioxidant capacity of the extracts was tested by DPPH assay, with good results, the cherry type variety having highest antioxidant effect. Pearson’s correlation coefficient showed some positive relationship between the antioxidant capacity and the total phenolic content, respectively the lutein content of the samples. 

Considering that tomato peels have lycopene, β-carotene, lutein, and different phenolic compounds in their composition, tomato industrial by-products could represent a source of natural bioactive molecules, at no additional costs, with applicability in nutraceuticals and food industry.

## Figures and Tables

**Table 1:** Content of individual carotenoids of ten tomato varieties peels extracts (mg/100 g DW) ± SD.

Sample	Carotenoids Content			
	Lycopene	β-Carotene	Lutein	Total Carotenoids
*Abellus*	1.77 ± 0.01	0.43 ± 0.01	0.26 ± 0.04	2.46 ± 0.07 ^bc^
*Aphen*	0.34 ± 0.05	0.38 ± 0.02	0.32 ± 0.02	1.04 ± 0.09 ^ab^
*Cristal*	1.30 ± 0.03	0.59 ± 0.02	0.60 ± 0.03	2.48 ± 0.07 ^bc^
*Lady rosa*	1.21 ± 0.02	0.33 ± 0.01	0.41 ± 0.02	1.95 ± 0.05 ^abc^
*Mirsini*	0.36 ± 0.03	0.16 ± 0.01	0.07 ± 0.01	0.60 ± 0.05 ^a^
*Rotunde mari*	2.80 ± 0.04	0.23 ± 0.01	0.31 ± 0.02	3.33 ± 0.07 ^c^
*Roșii lunguieţe*	1.38 ± 0.06	0.44 ± 0.01	0.46 ± 0.01	2.28 ± 0.07 ^bc^
*Ţărănești portocalii*	1.85 ± 0.02	0.24 ± 0.01	0.58 ± 0.02	2.67 ± 0.04 ^bc^
*Ţărănești roz*	3.70 ± 0.02	0.53 ± 0.01	1.09 ± 0.09	5.31 ± 0.12 ^d^
*Tiny Tim*	1.51 ± 0.03	0.45 ± 0.00	0.97 ± 0.02	2.93 ± 0.05 ^c^
Mean	1.62 ± 1.02	0.39 ± 0.14	0.51 ± 0.32	2.50 ± 1.29

Different letters indicate significant differences by Tukey’s test, at 5% probability.

**Table 2:** Individual concentrations of phenolic compounds and total phenolic content of ten tomato varieties peels extracts (mg/100 g DW) ± SD.

Sample	Content of Individual Phenolic Compounds						
	QTG	Q3R	di-CQA	tri-CQA	NGC	NG	Total Phenolic Content
*Abellus*	4.5 ± 0.0	11.9 ± 0.0	6.7 ± 0.1	5.0 ± 0.2	33.0 ± 0.3	4.6 ± 0.5	66 ± 1
*Aphen*	4.9 ± 0.1	7.7 ± 0.1	7.5 ± 0.0	5.2 ± 0.1	6.6 ± 0.7	4.7 ± 0.7	37 ± 2
*Cristal*	5.1 ± 0.1	10.4 ± 0.1	8.7 ± 0.2	6.5 ± 0.2	22.3 ± 0.7	7.1 ± 0.2	60 ± 1
*Lady rosa*	4.2 ± 0.1	9.5 ± 0.1	6.6 ± 0.3	5.4 ± 0.1	13.8 ± 0.2	4.7 ± 0.6	44 ± 1
*Mirsini*	5.4 ± 0.1	51.0 ± 0.1	8.9 ± 0.1	6.6 ± 0.2	70.6 ± 0.6	12.1 ± 0.4	155 ± 2
*Rotunde mari*	4.0 ± 0.1	6.2 ± 0.3	9.3 ± 0.4	6.4 ± 0.2	19.4 ± 0.9	4.8 ± 0.2	50 ± 2
*Roșii lunguieţe*	n. d.	8.7 ± 0.7	5.9 ± 0.1	5.9 ± 0.2	63.4 ± 0.2	10.6 ± 0.4	94 ± 2
*Ţărănești portocalii*	n. d.	6.4 ± 0.2	8.4 ± 0.6	5.1 ± 0.1	87.6 ± 1.2	12.9 ± 0.5	120 ± 3
*Ţărănești roz*	5.4 ± 0.1	11.4 ± 0.4	15.7 ± 0.2	8.9 ± 0.1	9.4 ± 0.2	6.7 ± 0.5	58 ± 2
*Tiny Tim*	3.6 ± 0.0	26.9 ± 1.0	8.6 ± 0.1	5.4 ± 0.1	22.8 ± 0.9	7.8 ± 0.3	75 ± 2
Mean	4.6 ± 0.7	18.1 ± 14.0	11.0 ± 2.7	6.9 ± 1.2	34.9 ± 28.5	7.6 ± 3.2	76 ± 2

QTG: quercetin-triglucoside; Q3R: quercetin-3-rutinoside (or rutin,); di-CQA: 3,4-di-O-caffeoylquinic acid (or isochlorogenic acid; tri-CQA: 3,4,5-tri-caffeoylquinic acid; NGC: naringenin chalcone; NG: naringenin.

**Table 3:** Minimum inhibitory concentration (mg tomato peels/mL) of the methanolic extracts.

Samples	G (+)			G(−)		
	*S. aureus*	*B. subtilis*	*L. monocitogenes*	*P. aeruginosa*	*E. coli*	*S. typhimurium*
*Abellus*	5.00	5.00	5.00	10.00	5.00	10.00
*Aphen*	5.00	10.00	10.00	10.00	5.00	10.00
*Cristal*	5.00	10.00	5.00	10.00	5.00	10.00
*Lady rosa*	5.00	5.00	<10.00	10.00	10.00	10.00
*Mirsini*	5.00	10.00	5.00	<10.00	10.00	<10.00
*Rotunde mari*	5.00	10.00	5.00	10.00	10.00	10.00
*Roşii lunguieţe*	5.00	5.00	5.00	10.00	10.00	10.00
*Ţărănești portocalii*	5.00	5.00	5.00	10.00	10.00	10.00
*Ţărănești roz*	2.50	2.50	5.00	10.00	10.00	10.00
*Tiny Tim*	5.00	10.00	10.00	<10.00	10.00	10.00
